# Integrated Transcriptomics and Metabolomics Reveal Key Insights into Iridoid Biosynthesis in *Gentiana crassicaulis* Seeds during Germination

**DOI:** 10.3390/genes15101255

**Published:** 2024-09-26

**Authors:** Lechen Xuan, Hongyang Xiao, Zhili Zhao, Jingxian Feng, Lianghong Ni, Jinrong Wu

**Affiliations:** School of Pharmacy, Shanghai University of Traditional Chinese Medicine, Shanghai 201203, China

**Keywords:** *Gentiana crassicaulis*, transcriptome, metabolome, iridoid biosynthetic pathway, seed germination process

## Abstract

**Background:** *Gentiana crassicaulis* Duthie ex Burk., a key species used in traditional Chinese medicine for treating rheumatic pain and stroke, contains iridoids as its primary active component. However, the biosynthetic mechanisms underlying iridoid production are not fully understood. **Methods:** This study focused on iridoid biosynthesis during the germination of *G. crassicaulis* seeds, integrating metabolomic and transcriptomic analyses to uncover the underlying pathways and key candidate genes. **Results:** 196,132 unigenes and 10 iridoid compounds were identified through RNA-seq and ultra performance liquid chromatography-quadrupole time of flight-mass spectrometer (UPLC-Q-TOF-MS), respectively. The intersection of results from Pearson correlation analysis and weighted gene co-expression network analysis (WGCNA) revealed a significant correlation between 26 genes and iridoid levels, suggesting their potential role in the iridoid metabolism. Notably, six highly expressed candidate genes (*DL7H*, *SLS*, *CYP76*, *CYP72A2*, *CYP84A1*, and *13-LOX3*) and five iridoids (loganic acid, sweroside, swertiamarin, gentiopicroside, and 6′-O-β-D-glucosyl-gentiopicroside) responded to methyl jasmonate stimulation in *G. crassicaulis* seedlings. **Conclusions:** by combining the known functions of candidate gene families, It is hypothesized that the *CYP716* and *LOX* families exert indirect influences on iridoid metabolism, while the *CYP71*, *CYP81*, *CYP72*, *CYP76*, *CYP710* families, *2OG-FeII* family, and the glucosyltransferase family are likely to play direct roles in the biosynthetic transformations of the five iridoids. This study provides a theoretical basis for further functional gene validation and metabolic engineering aimed at enhancing iridoid production. The insights gained could lead to improved iridoid production efficiency in medicinal plants, ultimately benefiting the quality and efficacy of medicinal materials.

## 1. Introduction

Iridoids, a distinct family of monoterpenoids [1], are characterised by their cyclic ether and hydroxyl groups. Structurally, they can be classified into secoiridoids and iridoids based on whether or not positions 7 and 8 of the cyclopentane ring are cleaved [2]. These compounds are widely distributed in medicinal plants, including *Catharanthus roseus* [3], *Camptotheca acuminata* [4], and *Rehmannia glutinosa* [5]. Iridoids are known for their diverse pharmacological activities, including hepatoprotective [6], choleretic, immunomodulatory [7], antitumor [8], and antioxidant effects [9], as well as their potential to treat diabetes and its complications [10].

The biosynthesis of iridoids is typically divided into three stages: intermediate synthesis, iridoid skeleton formation, and late modifications [11,12,13]. Iridoids originate from the common precursors isopentenyl diphosphate (IPP) and dimethylallyl diphosphate (DMAPP), which are produced through the mevalonic acid (MVA) and 2-C-methyl-D-erythritol-4-phosphate (MEP) pathways. IPP and DMAPP undergo a series of carbon chain rearrangements and redox reactions to form iridodial, the core skeleton of iridoids. Iridodial is then modified to produce various types of iridoids. Isotope labelling studies have demonstrated a sequential transformation relationship among sweroside, swertiamarin, and gentiopicroside, suggesting that these compounds are key metabolites in the iridoid synthesis pathway following loganic acid [14,15,16,17]. Current research is primarily focused on intermediate synthesis and iridoid skeleton formation, with studies on later modifications concentrated on the pathways upstream of loganic acid. However, the biosynthetic pathway following the cleavage of loganic acid remains unclear.

*Gentiana crassicaulis* Duthie ex Burk., primarily found in the Xizang, Yunnan, Sichuan, Guizhou, Qinghai, and Gansu regions of China [18], is an original plant of the commonly used traditional Chinese medicine “Qin Jiao” [19] (p. 282) and the Tibetan medicine “ཀྱི་ལྕེ།” (JieJi) [20]. It is known for its ability to relieve pain, clear dampness, and reduce heat [19] (p. 282). Extensive research on the chemical constituents of *G. crassicaulis* has revealed that its primary active compounds are iridoids, specifically loganic acid, sweroside, swertiamarin, and gentiopicroside [21]. These iridoids exhibit diverse pharmacological activities: loganic acid has anti-inflammatory [22], bone-preserving [23], and lipogenesis inhibiting activities [24]; sweroside offers liver protection [25] and neuroprotection, promotes osteogenesis, and has antioxidant and anti-glycemic effects [26]; swertiamarin is known for its hepatoprotective, hypoglycemic [27], and immunomodulatory effects [28,29], with therapeutic value in the treatment of various metabolic diseases [30]; and gentiopicroside is therapeutically beneficial for treating various diseases, including digestive tract diseases [31], tumours, neurological disorders [32], bone formation diseases [33], and inflammation [34]. These four iridoids are also found in other medicinal plants of the Gentianaceae family, such as *G. straminea* [21,35], *G. rigescens* [36], and *Swertia mileensis* [37]. Additionally, they are recognised as key components in the Chinese Pharmacopoeia, with loganic acid and gentiopicroside in “Qin Jiao”, gentiopicroside in “Long Dan”, sweroside in “Dang Yao”, and swertiamarin in “Qing Ye Dan” [19] (pp. 99, 140, 204, 282.).

Plant growth and development are accompanied by changes in secondary metabolites and gene expression [38,39,40]. Therefore, integrating transcriptomic and metabolomic data can enable the exploration of correlations between gene expression and metabolite fluctuations during plant development, laying the groundwork for identifying key genes in secondary metabolic pathways. In this study, an integrated analysis of the transcriptome and iridoid metabolome was conducted during the germination process of *G. crassicaulis* seeds to identify the candidate genes involved in iridoid biosynthesis. Elucidating these pathways will facilitate a deeper understanding of the mechanisms behind iridoid transformation in medicinal plants, offering theoretical support for research in plant breeding, medicinal plant resources, and synthetic biology.

## 2. Materials and Methods

### 2.1. Plant Materials

Seeds of *G. crassicaulis*. were collected in November 2022 from Ludian Township, Lijiang, Yunnan, China (latitude: 27°9′513″ N; longitude: 99°29′055″ E). The species identification was confirmed by Professor Lianghong Ni of Shanghai University of Traditional Chinese Medicine. Voucher specimens (No. 2022YN002) and seed samples are preserved in the specimen room of the Department of Pharmacognosy, School of Pharmacy, Shanghai University of Traditional Chinese Medicine.

### 2.2. Seed Preparation and Germination

The seeds were disinfected in 1% sodium hypochlorite solution for 15 min, followed by washing and soaking in distilled water. Subsequently, they were evenly spread on culture trays with ten layers of moist filter paper, with daily water supplementation to maintain moisture. The seeds were allowed to germinate in the dark in an incubator set at a constant temperature of 21 °C.

The first sample (Sample D0) was collected from soaked seeds. The second sample (Sample D9) was taken on the 9th day of germination, when more than half of the seed coats had ruptured, exposing the radicle. The third sampling (Sample D19) was collected on the 19th day, when more than half of the seeds had exposed cotyledons. The fourth sample (Sample D29) was taken on the 29th day, when germination had ceased. Each sample weighed approximately 0.5 g and was collected in nine replicates. Samples were stored at −80 °C. Three biological replicates were used for transcriptome sequencing, LC-MS analysis, and qRT-PCR.

### 2.3. Determination of Iridoids Using UPLC-Q-TOF-MS

To identify the iridoids, samples were first lyophilised to constant weight and ground into fine powder using liquid nitrogen. Approximately 0.1 g of the dried powder was accurately weighed and mixed with 10 mL of 80% methanol. This mixture was then sonicated for 30 min at 200 W and 53,000 Hz, cooled, shaken thoroughly, and filtered with 0.22 μm organic phase filtration membrane (Supin, Jiangsu, China) to obtain the LC-MS test samples [41]. Standard solutions of loganic acid, sweroside, swertiamarin, gentiopicroside, and 6′-O-β-D-glucosyl-gentiopicroside (Yanjin Biological, Shanghai, China) were prepared in methanol at concentrations of 0.740 mg/mL, 1.105 mg/mL, 1.120 mg/mL, 1 mg/mL, and 1.145 mg/mL, respectively.

For the LC analysis, a UPLC XB-C18 column (2.1 mm × 100 mm, 1.8 μm, Welch, SH, CHN) was used at 40 °C. The mobile phase consisted of 0.1% formic acid in water (A) and acetonitrile (B), with a gradient elution of 5% B (0–2 min), 5–12% B (2–5 min), 12–40% B (5–15 min), and 40% B (15–20 min), at a flow rate of 0.3 mL/min and an injection volume of 2 μL [21]. MS was conducted on an Agilent 6530 Accurate-Mass Q-TOF (Agilent, Santa Clara, CA, USA) with negative ion scanning. The electrospray ionization source was set with atomization pressure (GS1) at 55 psi, auxiliary pressure (GS2) at 55 psi, gas curtain pressure (CUR) at 35 psi, ion source temperature (TEM) at 550 °C, and spray voltage (IS) at 4 kV. The primary mass scan had a declustering voltage (DP) of 125 V, with a scan range from m/z 50 to 1700. The secondary mass scan utilised MS/MS spectrum acquisition in information-dependent acquisition (IDA) mode with a collision-induced dissociation (CID) energy of 30 V. Data analysis and peak area extraction were performed using Agilent MassHunter Qualitative Analysis B.06.00 software.

### 2.4. Transcriptome Analysis

Total RNA was extracted using a Trizol kit (Sangon, Shanghai, China) and purified using RNase-free DNase I (Sangon, Shanghai, China). Double-ended sequencing with 100 million reads per sample was performed on the Illumina NovaSeq 6000 platform (Illumina, San Diego, CA, USA). Sequencing services were provided by Sangon Biotechnology (Shanghai) Co., Ltd. (Shanghai, China).

The raw sequences were processed using Trimmomatic 0.36 [42] to remove low-quality reads. Subsequently, transcripts were de novo assembled using Trinity 2.0.6 [43] (parameters: min kmer cov 2). Transcripts ≥ 200 bp in length were clustered to minimise redundancy, and the longest sequence in each cluster was defined as a “unigene”. Pearson correlation analysis was performed on the unigenes of each sample using R package gplots 2.17.0. Subsequently, NCBI Blast+ 2.60 software [44] (e-value < 1 × 10^−5^) was used to align the unigene sequences against five databases: CDD, NR, NT, KOG (http://www.ncbi.nlm.nih.gov/COG/, accessed on 10 May 2023), and Pfam (http://pfam.xfam.org/, accessed on 10 May 2023) for functional annotation and classification. GO functional annotation and classification were performed using the GO database (https://geneontology.org/, accessed on 10 May 2023). Additionally, KAAS 2.1 software [45] was employed to compare the unigenes with the KEGG database (https://www.kegg.jp/, accessed on 10 May 2023) to analyse KEGG-related metabolic pathways. Gene expression levels were quantified as Transcript per million (TPM) values, with differential expression analysis performed using the R package DESeq 1.26.0. Unigenes with a q-value < 0.05 and |FoldChange| > 2 were classified as differentially expressed genes (DEGs). 

### 2.5. qRT-PCR Analysis

qRT-PCR was performed using the QuantStudio 3 system (Thermo Fisher, Waltham, MA, USA) with the qPCR Mix (2×, SYBR Green I, low ROX) kit (Bioleaper, Shanghai, China). Primers were designed using Primer Premier 5.0 software. RNA from the samples was extracted using a Total RNA Extractor (Trizol) kit (Sangon, Shanghai, China) and reverse-transcribed into cDNA using the MightyScript First Strand cDNA Synthesis Kit (BBI, Shanghai, China), with a concentration of 50 ng/uL. The PCR conditions were as follows: an initial denaturation at 95 °C for 2 min, followed by 40 cycles of 95 °C for 15 s, 60 °C for 30 s, and 95 °C for 15 s. The β-tubulin gene served as the reference gene. Relative gene expression levels were calculated using the 2^−ΔΔCt^ method [46]. Each gene was tested in triplicate for both biological and technical replicates. 

### 2.6. Candidate Gene Mining

Pearson correlation analysis was performed to explore the relationship between DEGs and iridoids. This analysis involved correlating the DEG expression matrix with the iridoid peak area matrix at different germination stages. Unigenes with correlation coefficients (*r*) ≥ 0.7 were considered to be highly correlated with iridoid metabolism.

For the weighted gene co-expression network analysis (WGCNA), genes with expression levels of 0 TPM in 8 or more of the 12 samples were excluded. The remaining genes were then used for the analysis. The co-expression network modules were constructed using the WGCNA package in R, with automatic network construction function (blockwiseModules) and default parameters: soft threshold power = 10, TOMtype = unsigned, mergeCutHeight = 0.25, and minModuleSize = 300. Pearson correlations between the identified modules and iridoid phenotypes were calculated. Modules with *r* > 0.7 were further analysed, and unigenes with both Gene Significance and Module Membership values >0.9 were designated as hub genes.

To identify candidate genes for the putative pathway from loganic acid to 6′-O-β-D-glucosyl-gentiopicroside [17], enzyme function keywords were assigned to each structural transformation step. Candidate genes were screened based on their high correlation with iridoids and hub gene status. Transcription factors and genes involved in primary metabolism were excluded from consideration. This approach ensured that only genes relevant to secondary metabolism were selected for further analysis. Candidate genes from hub genes and highly correlated DEGs were merged according to their gene families. These gene families were then mapped to the potential processes of the proposed pathway. The functional annotations for these processes were obtained using the KEGG-reaction database (https://www.kegg.jp/kegg/reaction/, accessed on 8 May 2024).

### 2.7. MeJA Treatment and Material Processing

The seeds mentioned in “Plant materials” were cultured on MS sterile medium for 7 months to produce seedlings. These seedlings were then uniformly sprayed with a 200 μmol/L solution of methyl jasmonate (MeJA) [47]. Leaf samples were collected at 0, 3, 6, and 12 h after treatment. Each leaf was longitudinally bisected along the veins, with one half used for HPLC analysis and the other for qRT-PCR. Three biological replicates were prepared for each time point, labelled MA, MB, and MC. The samples were immediately frozen in liquid nitrogen and stored at −80 °C.

For test sample preparation, the samples were lyophilised to constant weight, ground into fine powder, and accurately weighed to approximately 5 mg. This powder was then mixed with 200 μL of 80% methanol. Subsequently, this mixture was subjected to ultrasonic treatment for 30 min (200 W, 53,000 Hz), followed by centrifugation at 12,000 rpm for 15 min to collect the supernatant. This process was performed twice to ensure complete extraction, yielding the test solution.

The hybrid reference substance was prepared by mixing the standard solutions (prepared in methanol) of loganic acid, sweroside, swertiamarin, gentiopicroside, and 6′-O-β-D-glucosyl-gentiopicroside to obtain final concentrations of 0.099 mg/mL loganic acid, 0.155 mg/mL sweroside, 0.0504 mg/mL swertiamarin, 0.500 mg/mL gentiopicroside, and 0.041 mg/mL 6′-O-β-D-glucosyl-gentiopicroside in the hybrid reference substance.

The HPLC analysis was performed using an HPLC XB-C18 column (4.6 mm × 250 mm, 5 μm; Welch, Shanghai, China) and an Agilent 1260 High-Performance Liquid Chromatograph (Agilent, CA, USA). The column temperature was set at 30 °C, and the mobile phase comprised 0.04% formic acid (A) and acetonitrile (B). The gradient elution programme was as follows: 0–10 min at 9–11% B, 10–20 min at 11–12% B, and 20–30 min at 12% B. The flow rate was maintained at 0.8 mL/min, and the injection volume was 10 μL. Detection was achieved using a VWD detector at a wavelength of 240 nm [48].

### 2.8. Statistical Analysis

Statistical analysis of the iridoid peak area and content was performed using GraphPad Prism 9. Pearson correlation coefficient and significance levels were calculated using R 4.4.0. The pairwise two-tailed *t*-test was employed to determine statistical significance, with *p* < 0.05 considered statistically significant.

## 3. Results

### 3.1. Characterization and Enrichment Analysis of Iridoids during Seed Germination

**Figure 1:**
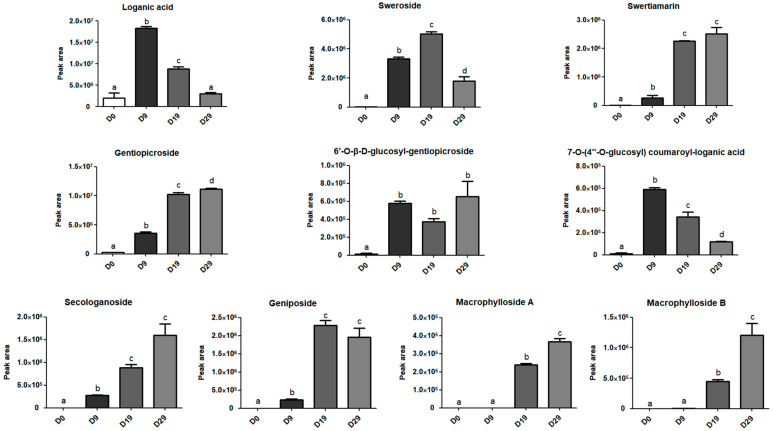
Relative contents of iridoids in *Gentiana crassicaulis* seeds at different times during germination, as determined by the peak area of chromatograms. Superscripts (a, b, c, and d) denote significant differences at *p* < 0.05. Data are presented as the mean ± standard error of the mean (SEM) of *n* = 3 replicates. D0, D9, D19, and D29 correspond to the four sampling times during the seed germination of *G. crassicaulis*.

Ten iridoids were identified during the seed germination of *G. crassicaulis* through comparisons with reference substances, mass spectrometry data, and literature sources (Table S1). To evaluate changes in iridoid levels at four different stages of seed germination, cluster analysis and principal component analysis (PCA) were performed using the mass spectrum peak areas of the 10 iridoids. The results showed that all samples were clustered into four different groups, with each stage of germination forming its own cluster. Principal components PC1 (61.5%) and PC2 (29.3%) together accounted for 90.8% of the total variance, indicating that the principal components capture most of the variability in the data. This suggests that the biological replicates are consistent and that the data are robust for further analysis (Figure S1A,B). Seven iridoids were detected in the D0 sample, excluding geniposide, macrophylloside B, and macrophylloside A. In the D9 sample, nine iridoids were detected, excluding macrophylloside A. Furthermore, all ten iridoids were detected in both the D19 and D29 samples (Figure S1C). The peak areas of the different iridoids showed varying trends at different stages. From D0 to D9, there was a significant increase in the peak areas of all iridoids, except for macrophylloside B and macrophylloside A. During the period from D9 to D19, the peak areas of loganic acid (*p* = 0.0001) and 7-O-(4″-O-glucosyl) coumaroyl-loganic acid (*p* = 0.0069) decreased significantly, while those of the other iridoids, except for 6′-O-β-D-glucosyl-gentiopicroside, increased significantly. During the period from D19 to D29, the peak areas of 7-O-(4″-O-glucosyl) coumaroyl-loganic acid (*p* = 0.0006) and sweroside (*p* = 0.0082) decreased significantly, whereas those of gentiopicroside (*p* = 0.0480), macrophylloside B (*p* = 0.0183), and macrophylloside A (*p* = 0.0029) increased significantly. Throughout the germination process of *G. crassicaulis* seeds, the decrease in the peak area of loganic acid occurred earliest. Meanwhile, sweroside began to accumulate earlier than swertiamarin, and sweroside was consumed later than loganic acid. Structurally, sweroside is more similar to swertiamarin and gentiopicroside, but its trend in peak area changes is similar to that of loganic acid (Figure 1).

### 3.2. Functional Annotation of Unigenes

A total of 176.79 GB of sequence data were generated from 12 samples. After assembling valid reads, a total of 196,132 unigenes were obtained, ranging in length from 201 bp to 17,031 bp, with an average length of 653 bp; 32,942 unigenes (16.79%) were ≥1000 bp (Figure S2A). Comparison with CDD, NR, NT, GO, KEGG, Pfam, and KOG databases revealed that the NR database provided the most annotations, covering 81,988 unigenes. Overall, 96,069 unigenes were annotated in at least one database, accounting for 48.98% of all unigenes. The annotation results are summarised in Figure S2B. Correlation analysis of unigene expression revealed that the correlations within each group were ≥0.79, whereas the correlations between groups were ≤0.76, indicating that the correlation within each group was greater than that between groups and that each group was clearly distinguishable (Figure S2C).

To understand the functional classification of unigenes, 44,019 annotated unigenes were analysed in the GO database. GO functions can be categorised into biological processes, cellular components, and molecular functions. Enrichment analysis was performed on the top 10 annotated items in each category (Figure S2D). Notably, the most highly represented categories were “metabolic process” (6130 unigenes) and “catalytic activity” (4250 unigenes). To further elucidate the metabolic pathways involving the unigenes, the KEGG secondary metabolic pathways associated with them were analysed. A total of 2716 unigenes were found to participate in 47 standard pathways of KEGG secondary metabolism. The top 10 pathways are summarised in Table 1. The phenylpropanoid biosynthesis pathway had the highest number of associated unigenes (328). Among them, the pathways related to iridoid—“terpenoid backbone biosynthesis” (226 unigenes) and “ubiquinone and other terpenoid biosynthesis” (156 unigenes)—ranked second and fifth, respectively. These findings suggest that *G. crassicaulis* seeds exhibit vigorous metabolic activity during germination, with a significant number of genes involved in terpenoid metabolism, making it a promising system for mining genes related to iridoid metabolism.

**Figure 2:**
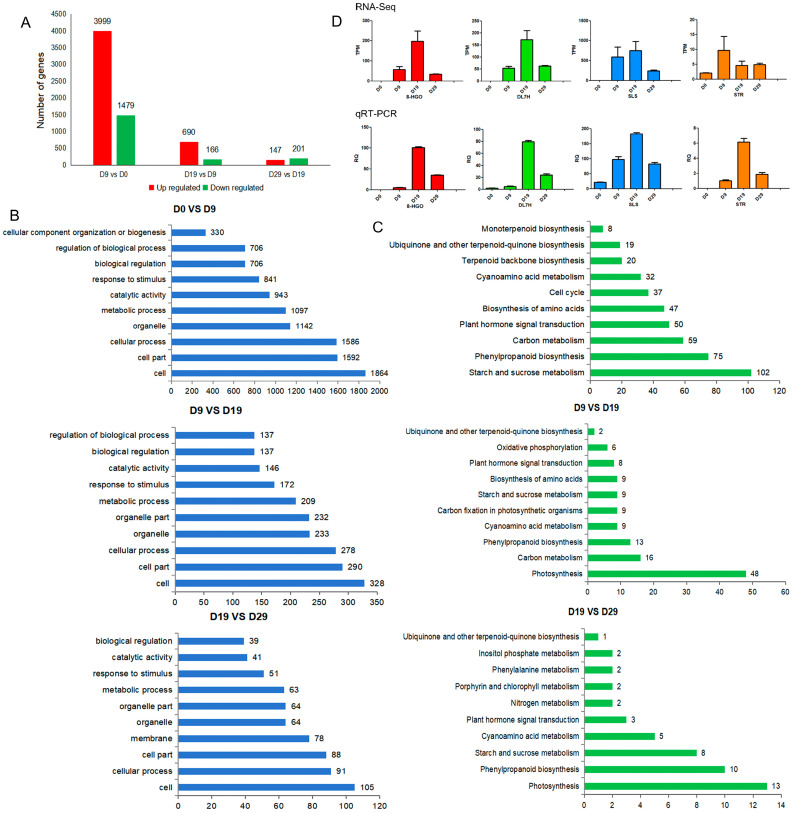
Differentially expressed gene (DEG) analysis and qRT-PCR validation. (**A**) Number of DEGs identified through comparisons between different germination stages. (**B**) Distribution of DEGs across the top 10 enriched GO terms. (**C**) Distribution of DEGs across the top 10 enriched KEGG pathways. (**D**) qRT-PCR analysis of four enzyme-encoding genes (*8-HGO*, *DL7H*, *SLS*, and *STR*) involved in iridoid biosynthesis. The relative expression levels of the genes were normalised against the β-tubulin gene as an internal control, with D0 set as the reference point. Data are presented as the mean ± SEM of *n* = 3 replicates.

To investigate the genetic differences across various germination stages, DEG analysis was conducted. D0 vs. D9, D9 vs. D19, and D19 vs. D29 comparisons revealed a total of 6153 DEGs. The expression patterns of these DEGs in each sample are illustrated in Figure S3. As shown in Figure 2A, the D0 vs. D9 comparison yielded the most DEGs, with 3999 up-regulated and 1479 down-regulated unigenes. The D9 vs. D19 comparison yielded 690 up-regulated and 166 down-regulated unigenes, while the D19 vs. D29 comparison revealed 147 up-regulated and 201 down-regulated unigenes. To elucidate the biological functions of DEGs at different germination stages, GO enrichment analysis was performed. The terms “cell”, “cellular process”, and “cell part” were consistently enriched in the top three positions across different germination stages. Additionally, “catalytic activity” and “metabolic process” were consistently enriched in the top ten categories. These findings suggest that the internal environment during seed germination plays a key role in the biotransformation of iridoid (Figure 2B).

Additionally, KEGG pathway enrichment analysis was conducted to identify the metabolic pathways associated with DEGs. In the D0 vs. D9 comparison, 20 unigenes were associated with “terpenoid backbone biosynthesis”, 19 unigenes with “ubiquinone and other terpenoid-quinone biosynthesis”, and 8 unigenes with “monoterpene biosynthesis”. However, in the D9 vs. D19 and D19 vs. D29 comparisons, only one unigene was enriched in “ubiquinone and other terpenoid-quinone biosynthesis” (Figure 2C).

### 3.3. qRT-PCR Validation

To confirm the accuracy of the transcriptome data, four enzyme-encoding genes (*8-HGO*, *DL7H*, *SLS*, and *STR*) involved in the iridoid biosynthesis pathway were selected for further analysis. Their expression levels were measured at four stages of seed germination using qRT-PCR (Figure 2D). The relative expression levels of these genes showed an initial increase followed by a decrease as germination progressed. The qRT-PCR results were consistent with the transcriptome data, thereby validating the reliability of the transcriptomic analysis.

### 3.4. Correlation Analysis of Transcriptome and Metabolome

**Figure 3:**
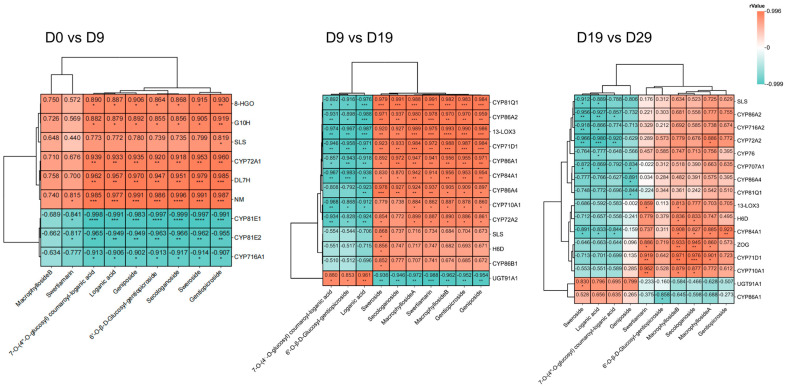
Cluster heatmap of correlations between highly correlated DEGs and iridoids in the D0 vs. D9, D9 vs. D19, and D19 vs. D29 comparisons. Red and blue indicate positive and negative correlations, respectively. Statistical significance is indicated as * *p* < 0.05, ** *p* < 0.01, *** *p* < 0.001, and **** *p* < 0.0001.

Correlation analysis between the DEGs and iridoids revealed several significant relationships (Figure 3). In the D0 vs. D9 comparison, nine candidate genes were identified. Among these, genes such as *G10H*, *8-HGO*, *DL7H*, *NM*, and *SLS*, along with one *CYP72A1*, exhibited positive correlations with iridoids, suggesting their involvement in iridoid biosynthesis. Conversely, one *CYP716A1* and two *CYP81E*s were negatively correlated with iridoids. Among these, *G10H*, *8-HGO*, *SLS*, *DL7H*, and *NM* encode enzymes known to be involved in the iridoid biosynthesis pathway. In the D9 vs. D19 comparison, 13 candidate genes were identified. Of these, ten *CYP*s, one *13-LOX3*, and one hyoscyamine 6-dioxygenase (*H6D*) gene were negatively correlated with three iridoids and positively correlated with seven others. Additionally, one *UGT* was positively correlated with three iridoids and negatively correlated with seven iridoids. In the D19 vs. D29 comparison, 16 candidate genes were obtained, of which six *CYP*s, one *13-LOX3*, and one *H6D* gene were positively correlated with six iridoids and negatively correlated with four iridoids. Three *CYP*s were positively correlated with five iridoids and negatively correlated with five iridoids. Two *CYP*s and one *ZOG* were positively correlated with seven iridoids and negatively correlated with three others. Furthermore, one *UGT91A1* and one *CYP86A1* were positively correlated with four iridoids and negatively correlated with six iridoids. In total, 26 candidate genes were identified across the D0 vs. D9, D9 vs. D19, and D19 vs. D29 comparisons. Of these, nineteen belonged to the P450 family, one *H6D* gene belonged to the *2OG-FeII* family, one *13-LOX3* gene belonged to the *LOX* lipoxygenase family, and both *UGT91A1* and *ZOG* belonged to the *UGT* family.

**Figure 4:**
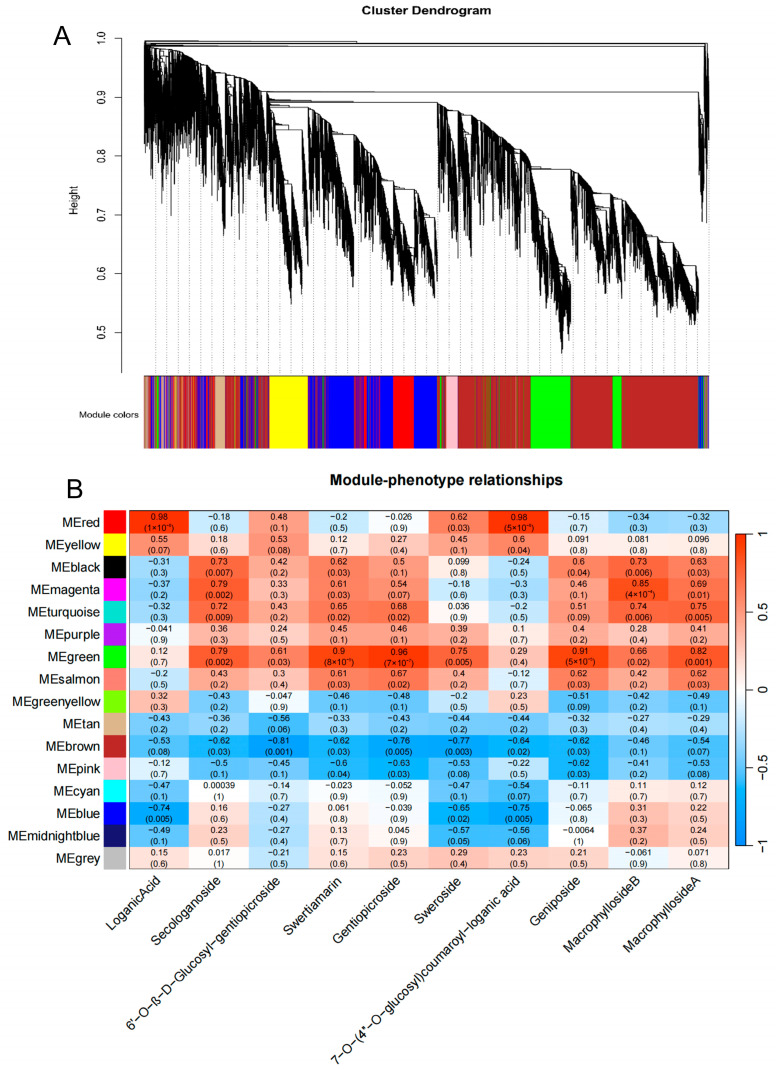
Weighted gene co-expression network analysis (WGCNA) of 88,098 unigenes. (**A**) Hierarchical clustering tree (cluster dendrogram) displaying the 16 co-expressed gene modules identified through WGCNA. (**B**) Distribution of genes across modules and the correlation between each module and the 10 iridoids. The Pearson correlation coefficient and the e-value are indicated in the grid for each module and trait.

WGCNA categorised 88,098 genes into 16 modules based on their expression patterns (Figure 4A). Notably, the “MEred” module showed a significant positive correlation with loganic acid and 7-O-(4″-O-glucosyl) coumaroyl-loganic acid, whereas the “MEblue” module showed a significant negative correlation with these two components. The “MEBlack” and “MEmagenta” modules were positively correlated with secologanoside and macrophylloside B, while three iridoids were positively correlated with the “MEturquoise” module. The “MEgreen” module was positively correlated with six iridoids, while the “MEbrown” module was negatively correlated with three iridoids. Specifically, the “Mered” and “MEblue” modules are associated with loganic acid, the “MEgreen” module is associated with swertiamarin/gentiopicroside/sweroside, and the “MEbrown” module is associated with 6′-O-β-D-glucosyl-geniopicroside/gentiopicroside/sweroside (Figure 4B). These modules contain hub genes that are potentially involved in the metabolism of iridoids, including unigenes annotated as members of the *CYP71*/*81*/*84*/*86*/*72*/*76*/*707* family, the *LOX* lipoxygenase family, the *2OG-FeII* family, and the glycosyltransferase family. The findings from the WGCNA and DEG correlation analysis corroborate each other, validating the observed correlations.

### 3.5. Response of Candidate Genes and Iridoids to MeJA Stimulation

To elucidate the function of candidate genes, six candidate genes with a TPM > 10 in samples and five key iridoids (loganic acid, sweroside, swertiamarin, gentiopicroside, and 6′-O-β-D-glucosyl-gentiopicroside) were selected and subjected to MeJA hormone stimulation in *G. crassicaulis* seedlings. Following MeJA treatment, three sterile *G. crassicaulis* seedlings exhibited varying degrees of decrease in loganic acid levels and an increase in the levels of four other secoiridoids within 12 h post-treatment (Figure S4A). The expression of *DL7H* showed a consistent decrease; *SLS* expression initially increased and then decreased in samples MA and MB, while it only increased in sample MC. The expression of *13-LOX3* initially increased and then decreased in all three samples. *CYP76* and *CYP72A2* expressions showed a pattern of initial decrease followed by an increase across all samples, whereas *CYP84A1* expression varied, with some samples showing an increase (Figure S4B).

**Figure 5:**
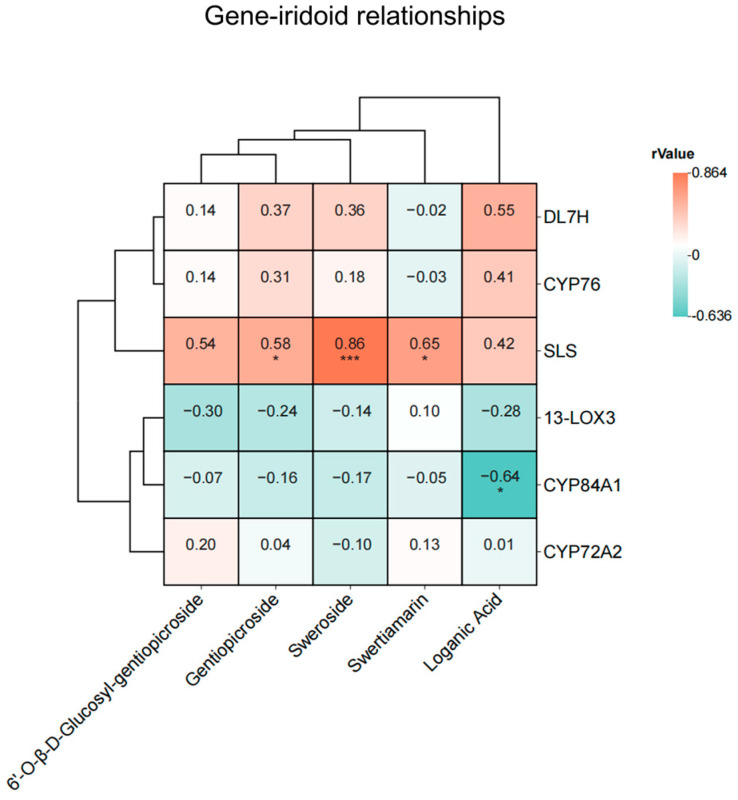
Pearson correlation analysis of iridoid contents and the relative expression of candidate genes following MeJA stimulation in aseptic *Gentiana crassicaulis* seedlings. Statistical significance is indicated as * *p* < 0.05 and *** *p* < 0.001.

To further investigate the relationship between gene expression changes and iridoid contents in *G. crassicaulis* seedlings following MeJA stimulation, a Pearson correlation analysis was conducted between the levels of five iridoids and the relative expression of the six candidate genes (Figure 5). *SLS* showed a positive correlation with sweroside, swertiamarin, and gentiopicroside, with correlation coefficients (*r*) of 0.86, 0.65, and 0.58, respectively. Conversely, *CYP84A1* exhibited a negative correlation with loganic acid (*r* = −0.64).

## 4. Discussion

**Figure 6:**
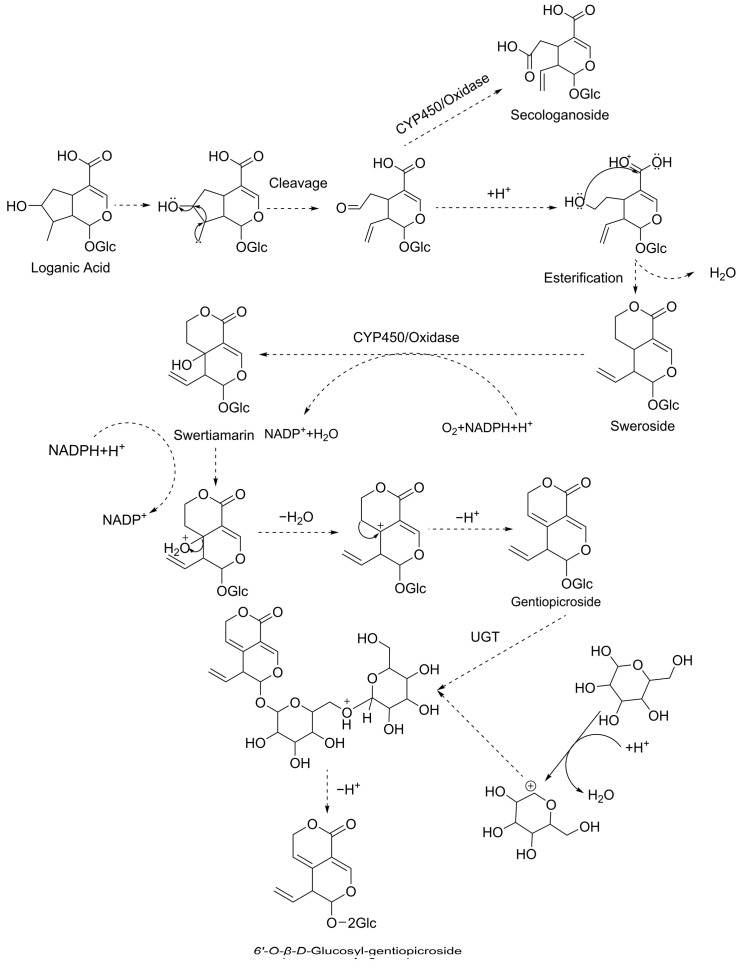
Proposed biosynthetic pathway from loganic acid to 6′-O-β-D-glucosyl-gentiopicroside. Initially, loganic acid is cleaved at positions 7 and 8, followed by esterification at positions 7 and 11, forming sweroside. Sweroside is then oxidised and hydroxylated at position 5 to form swertiamarin. Swertiamarin undergoes dehydroxylation at position 5, forming a double bond between positions 5 and 6, which leads to the formation of gentiopicroside. Finally, gentiopicroside is converted into 6′-O-β-D-glucosyl-gentiopicroside through the action of a glucosyltransferase (*UGT*).

Foundational research has elucidated the biosynthesis pathway of iridoids in plants, highlighting the transformations and interrelationships among key iridoid compounds. C-14 isotope labelling studies have clarified the sequential metabolic interconversion of sweroside, swertiamarin, and gentiopicroside. Coscia et al. [14] extracted loganic acid and gentiopicroside with C-14 following C-14 labelling of MVA in *Sambucus carolineinsis*. Similarly, Inouye et al. [15] detected C-14 in both sweroside and swertiamarin using C-14-labelled MVA in *Scutellaria japonica*, with sweroside exhibiting a 10-fold higher C-14 incorporation compared to swertiamarin. They also successfully labelled sweroside with C-14 in *G. scabra*, leading to the detection of C-14 in gentiopicroside [16]. Building upon these findings, Jensen et al. [17] summarised a series of experiments and proposed a sequential transformation relationship between loganic acid, sweroside, swertiamarin, and gentiopicroside in plants. The present study corroborates this sequential transformation relationship, as evidenced by the accumulation patterns of these iridoids during seed germination. Guided by these outcomes and common patterns of chemical structural change, we have further hypothesised the biosynthetic pathway and the mechanism of transformation from loganic acid to 6′-O-β-D-glucosyl-gentiopicroside, considering the general patterns of structural changes (Figure 6). Loganic acid undergoes a series of reactions, including cleavage at positions 7 and 8 and esterification at positions 7 and 11, producing various intermediates and ultimately forming sweroside. Sweroside is then oxidised and hydroxylated at position 5 to form swertiamarin. Swertiamarin undergoes dehydroxylation at position 5, forming a double bond between positions 5 and 6, yielding gentiopicroside. Finally, gentiopicroside is converted into 6′-O-β-D-glucosyl-gentiopicroside. 

**Figure 7:**
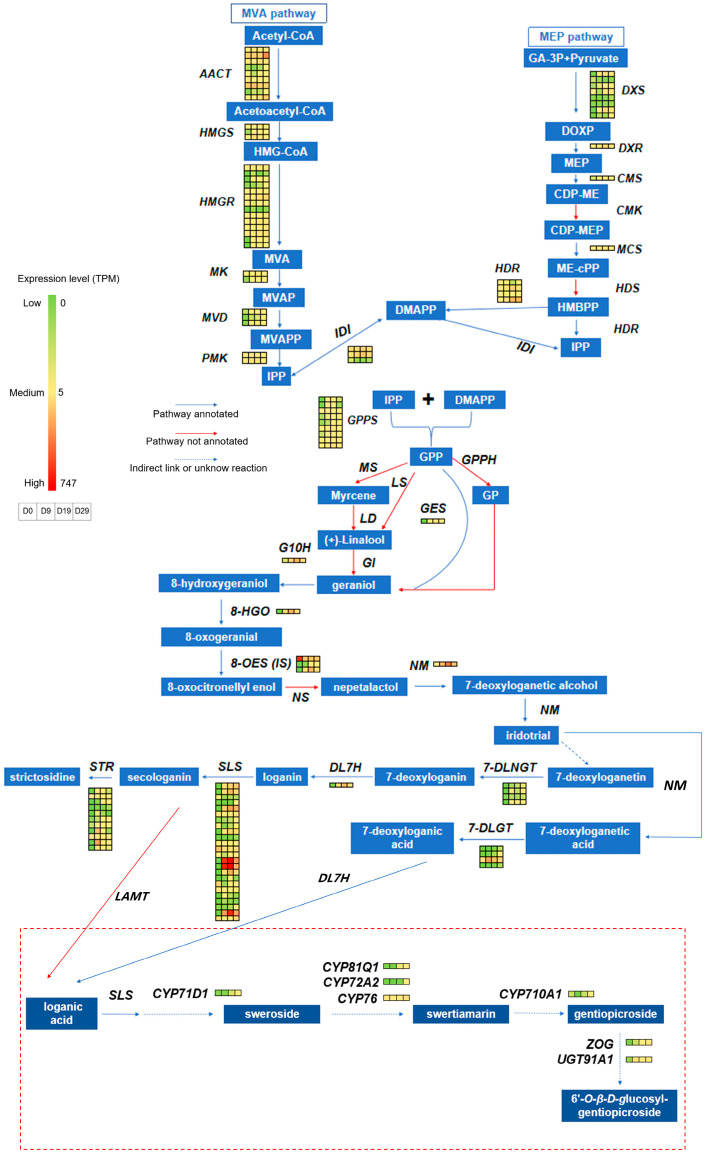
Expression of genes involved in iridoid biosynthesis. The red dashed box highlights the putative pathways and candidate genes.

Combining transcriptome annotations with candidate gene analysis, we hypothesised the involvement of several key gene families in the iridoid biosynthetic pathway (Figure 7). The *H6D* gene, belonging to the *2OG-FeII* family [49], is potentially involved in multiple stages of iridoid metabolism. Members of the *CYP71* family are hypothesised to participate in the formation of sweroside and swertiamarin. The *CYP76* family is implicated in forming swertiamarin. The *CYP710* family is potentially implicated in the formation of gentiopicroside. The *CYP81* family is suggested to play a role in the modification of secoiridoids. The *SLS* gene, belonging to the *CYP72* family, may be crucial for sweroside formation, and the *CYP716* and *LOX* families may indirectly affect iridoid metabolism. These inferences need confirmation through subsequent in vivo and in vitro functional validation experiments, and to ensure their universality in iridoid metabolism future studies should extend these validations across multiple plant species.

Members of the *2OG-FeII* family catalyse a broad spectrum of oxidation reactions in plants [50], including hydroxylation, demethylation, ring formation, rearrangement, desaturation, and halogenation, suggesting their involvement in multiple stages of iridoid metabolism. Members of the *CYP71* family are extensively implicated in the oxidation of diverse terpenoids, including processes such as hydroxylation and the formation of oxygen-containing rings [51,52]. These family members are hypothesised to participate in the formation of the sweroside lactonic ring or the hydroxylation of swertiamarin at position 5. The *CYP76* family, known for its role in the oxidation of monoterpenoids and diterpenoids, includes the *G10H* gene, which encodes *CYP76B*. *CYP76 H/M* is associated with the hydroxylation of the bridgehead carbon at position 9 of tricyclic diterpenes [53]. Additionally, a *CYP76* enzyme has been found to be negatively correlated with sweroside and positively correlated with swertiamarin during seed germination, suggesting its role in the hydroxylation of the bridgehead carbon at position 5 of sweroside to form swertiamarin. Furthermore, *CYP710* family members are reported to encode sterol C22 desaturases, enzymes that facilitate the formation of double bonds [54]. *CYP710* shows a positive correlation with gentiopicroside during seed germination, indicating its potential role in the formation of the double bond between positions 5 and 6 in gentiopicroside. *CYP81* family members are known for their role in the ring oxidation modification of flavonoids and naphthalene compounds [55,56]. During seed germination, *CYP81* demonstrated a significant correlation with gentiopicroside, swertiamarin, and other iridoids, suggesting its role in the modification of secoiridoids. The *SLS* gene, belonging to the *CYP72* family, is a key enzyme in the cleavage of iridoids at positions 7 and 8 in *C. acuminata* and *C. roseus* [11]. It exhibited a significant positive correlation with sweroside during seed germination and in response to MeJA hormone stimulation in sterile seedlings.

The *CYP716* family of enzymes is specifically involved in the oxidative modification of the triterpenoid skeleton [57]. IPP and DMAPP serve as common precursors for both iridoids and triterpenoids [58]. These precursors are converted into geranyl diphosphate (GPP) for monoterpenoid synthesis, whereas the formation of farnesyl diphosphate (FPP) indicates a pathway towards triterpenoid synthesis. *CYP716* enzymes catalyse the oxidation of β-amyrinol, an FPP product, and this process may involve the consumption of iridoid precursors IPP and DMAPP. This mechanism potentially explains the observed negative correlation between *CYP716* expression and iridoid levels. The *13-LOX* enzyme catalyses the oxidation of linoleic or linolenic acid at the 13th position, leading to the formation of 13-hydroperoxide fatty acids [59]. These 13-hydroperoxide fatty acids undergo a series of reactions that result in the production of jasmonic acid [60]. Jasmonic acid is an endogenous plant hormone known for its broad impact on secondary metabolic pathways, and its influence on the iridoid pathway has been documented [61,62]. Members of the *13-LOX* family likely influence the metabolism of iridoids by modulating jasmonic acid synthesis.

## 5. Conclusions

This study identified candidate genes potentially influencing iridoid metabolism through combined transcriptome and metabolome analyses during seed germination in *G. crassicaulis*. Key genes from the *CYP71*, *CYP81*, *CYP84*, *CYP86*, *CYP72*, *CYP76*, *CYP707*, and *CYP710* families, as well as those from the *LOX* lipoxygenase family, *2OG-FeII* family, and glycosyltransferase family, were implicated. While *CYP716* and *LOX* families may have indirect effects on iridoid metabolism, the *CYP71*, *CYP81*, *CYP72*, *CYP76*, and *CYP710* families; the *2OG-FeII* family; and the glucosyltransferase family are likely involved in the transformation of the five iridoids (loganic acid, sweroside, swertiamarin, gentiopicroside, and 6′-O-β-D-glucosyl-gentiopicroside). These findings provide a basis for further research into the plant iridoid biosynthetic pathways, offering insights into the potential application of metabolic engineering to optimise iridoid production. Moreover, they furnish molecular-level evidence that substantiates the pharmacological foundation of the traditional Chinese medicine “Qinjiao”.

## Figures and Tables

**Table 1:** Top 10 KEGG secondary metabolic pathways identified through the analysis of transcriptomic unigenes.

No.	KEGG Secondary Metabolic Pathways	Pathway ID	No. of Unigenes	Percentage
1	Phenylpropanoid biosynthesis	ko00940	328	12.08%
2	Terpenoid backbone biosynthesis	ko00900	226	8.32%
3	Porphyrin and chlorophyll metabolism	ko00860	196	7.22%
4	Ubiquinone and other terpenoid-quinone biosynthesis	ko00130	156	5.74%
5	One carbon pool by folate	ko00670	153	5.63%
6	Nicotinate and nicotinamide metabolism	ko00760	133	4.90%
7	Pantothenate and CoA biosynthesis	ko00700	115	4.23%
8	Folate biosynthesis	ko00790	102	3.76%
9	Isoquinoline alkaloid biosynthesis	ko00950	99	3.65%
10	Tropane, piperidine, and pyridine alkaloid biosynthesis	ko00960	77	2.84%
	47 secondary metabolic pathways in all		2716	

## Data Availability

The raw sequence data generated in this study have been deposited in the Short Read Archive (SRA) of the NCBI under accession number PRJNA1141215 (https://dataview.ncbi.nlm.nih.gov/object/PRJNA1141215, accessed on 29 July 2024).

## Supplementary Materials

The following supporting information can be downloaded at: https://www.mdpi.com/article/10.3390/genes15101255/s1, Figure S1: Analysis of iridoid metabolites, Figure S2: Quality assessment of transcriptome during seed germination of *Gentiana crassicaulis*, Figure S3: Heat map showing the expression trends of differentially expressed gene across all samples, Figure S4: Analysis of iridoid contents and the relative expression of candidate genes following MeJA stimulation in aseptic *Gentiana crassicaulis* seedlings, Table S1: Summary of iridoid spectral information, Table S2: Primer list used in qRT-PCR analysis.

## Abbreviations

IPP: Isopentenyl diphosphate; DMAPP: Dimethylallyl diphosphate; MVA: Mevalonate; MEP: Methylerythritol phosphate; *DL7H*: 7-Deoxyloganetic acid; *8-HGO*: 8-hydroxygeraniol oxidoreductase; *SLS*: Secologanin synthase; *H6D*: hyoscyamine 6-dioxygenase; *LOX*: Lipoxygenase; *UGT*: Uridine diphosphate glycosyltransferases; GO: Gene ontology; KEGG: Kyoto Encyclopedia of Genes and Genomes; CDD: Conserved Domains Database; NR: Non-Redundant Protein Sequence Database; NT: Nucleotide Sequence Database; Pfam: The protein families database; KOG: Eukaryotic Orthologous Groups; DEGs: Differentially expressed genes; UPLC-Q-TOF-MS: Ultra Performance Liquid Chromatography-Quadrupole Time of Flight-Mass Spectrometer; RNA-seq: RNA sequencing; HPLC: High Performance Liquid Chromatography; TPM: Transcript per million; PCA: Principal component analysis; PCR: Polymerase chain reaction; qRT-PCR: Quantitative real time polymerase chain reaction; WGCNA: weighted gene co-expression network analysis.
